# Immunotherapy resistance and strategies in malignant pleural mesothelioma

**DOI:** 10.20517/cdr.2025.215

**Published:** 2026-03-26

**Authors:** Wanyi Xia, Yulong Zhang, Juanzhi Zhao, Xiaoli Tan, Shaolong Ju, Wei Zou, Chaoqun Chen, Chunwei Li, Yanghua Xu, Yingming Peng, Shengqiao Li

**Affiliations:** ^1^Cancer Center, The Fifth Affiliated Hospital of Sun Yat-sen University, Zhuhai 519000, Guangdong, China.; ^2^Clinical Pharmaceutics Room, The Fifth Affiliated Hospital of Sun Yat-sen University, Zhuhai 519000, Guangdong, China.; ^3^Experimental Research Department, The Fifth Affiliated Hospital of Sun Yat-sen University, Zhuhai 519000, Guangdong, China.; ^4^Center for Infection and Immunity Research, The Fifth Affiliated Hospital of Sun Yat-sen University, Zhuhai 519000, Guangdong, China.; ^5^Clinical Lab, The Fifth Affiliated Hospital of Sun Yat-sen University, Zhuhai 519000, Guangdong, China.

**Keywords:** Malignant pleural mesothelioma, immunotherapy, resistance to immune checkpoint inhibitors, tumor microenvironment

## Abstract

Malignant pleural mesothelioma (MPM) remains one of the most aggressive thoracic malignancies, characterized by profound resistance to conventional modalities such as surgery, chemotherapy, and radiotherapy, resulting in persistently poor survival outcomes. The advent of immune checkpoint inhibitors (ICIs) has fundamentally reshaped the therapeutic landscape of MPM. Notably, dual programmed cell death protein 1 (PD-1)/cytotoxic T-lymphocyte-associated protein 4 (CTLA-4) blockade has demonstrated superior efficacy over monotherapy in multiple phase I/II trials and has been established as a novel first-line standard of care. Nevertheless, the high incidence of resistance continues to pose a major clinical challenge. This therapeutic bottleneck is largely attributed to the unique biology of MPM, including a profoundly immunosuppressive tumor microenvironment, aberrantly activated signaling pathways, and complex metabolic reprogramming, which together form a multilayered defense network against immune attack. In response to this intricate resistance architecture, recent research efforts have increasingly focused on the development of precision combination strategies. By rationally integrating ICIs with anti-angiogenic agents, chemotherapy, metabolic modulators, and next-generation cellular immunotherapies [e.g., chimeric antigen receptor T cells (CAR-T), chimeric antigen receptor-natural killer (CAR-NK)], these approaches aim to dismantle immune evasion barriers and reinvigorate antitumor immunity. Concurrently, the discovery of novel biomarkers and their integration with multi-omics data are enabling more precise patient stratification, signaling the advent of an era of personalized immunotherapy for MPM. This review provides a systematic synthesis of the latest clinical advances and fundamental breakthroughs in MPM immunotherapy, with a particular focus on dissecting the multifactorial mechanisms underlying therapeutic resistance. Its core contribution lies in constructing a forward-looking framework for next-generation treatment strategies. It critically evaluates the translational potential of emerging approaches, including arginine deprivation therapy for argininosuccinate synthase 1 (ASS1)-deficient tumors, CAR-T cells, T-cell receptor fusion constructs, and oncolytic virotherapy. By integrating these innovative modalities with biomarker-guided patient selection, this review delineates a roadmap for transitioning MPM management from empirical therapy toward precision immuno-oncology, with the ultimate goal of achieving durable disease control in this challenging malignancy.

## INTRODUCTION

Malignant pleural mesothelioma (MPM) is an aggressive and lethal malignancy arising from the mesothelial cells of the pleural cavity, most commonly linked to asbestos exposure^[1]^. Despite advances in understanding its pathogenesis, the overall prognosis remains dismal, with median overall survival (OS) reportedly limited to 12-18 months under conventional therapies^[2]^. The global incidence, though declining in several developed countries due to asbestos regulation, continues to rise in developing regions where industrial use persists, reflecting the disease’s long latency period of 30-50 years^[3,4]^.

For nearly two decades, the standard first-line regimen for unresectable MPM consisted of platinum-based chemotherapy combined with pemetrexed, as established in the pivotal 2003 Vogelzang trial. However, despite modest improvements in progression-free survival (PFS) and symptom control, long-term outcomes plateaued, and second-line options offered minimal benefit. Multimodal approaches integrating surgery and radiotherapy were limited to selected patients, with high recurrence rates and substantial morbidity^[5,6]^.

The emergence of immune checkpoint inhibitors (ICIs) has substantially reshaped the therapeutic paradigm for MPM, although their clinical benefit remains heterogeneous across patient subgroups. The CheckMate 743 phase III trial represented a turning point, demonstrating a significant OS benefit for nivolumab plus ipilimumab over standard chemotherapy [median OS 18.1 *vs.* 14.1 months; hazard ratio (HR) ≈ 0.73], particularly among patients with non-epithelioid histology. Subsequent studies such as KEYNOTE-483 (NCT02784171) and BEAT-meso (NCT03762018) confirmed the value of immunotherapy either as monotherapy or in combination with chemotherapy and anti-angiogenic agents, leading to the formal recognition of dual ICI therapy as a standard first-line treatment for unresectable MPM^[7-9]^.

Nevertheless, not all patients benefit from ICIs, and a considerable fraction exhibits primary or acquired resistance. Tumor heterogeneity, a profoundly immunosuppressive microenvironment, and a low mutational burden collectively contribute to objective response rates (ORRs) - typically < 25% for monotherapy. Moreover, the absence of validated predictive biomarkers and the emergence of immune-related adverse events (irAEs) complicate clinical management^[10-12]^.

In this context, research in MPM has rapidly shifted from establishing immunotherapy efficacy toward understanding and overcoming resistance. Current investigations aim to elucidate the molecular and cellular determinants of immune evasion, identify predictive biomarkers, and design rational combination strategies that enhance immune responsiveness. This review provides a comprehensive overview of the immunopathogenesis of MPM, clinical advances in immunotherapy, mechanisms of therapeutic resistance, and emerging strategies - including metabolic targeting, cellular immunotherapies, and oncolytic platforms - that are poised to redefine the management of this challenging malignancy^[13-16]^.

## IMMUNOPATHOGENESIS AND TUMOR MICROENVIRONMENT: MOLECULAR AND IMMUNOLOGIC BASIS OF MESOTHELIOMA

Asbestos exposure, responsible for nearly 80% of MPM, initiates a decades-long cascade of chronic inflammation and oxidative injury^[17]^. Biopersistent fibers lodged in the pleura trigger frustrated phagocytosis, generating persistent reactive oxygen species (ROS)/reactive nitrogen species (RNS), DNA damage, chromosomal instability, and release of pro-tumorigenic cytokines [tumor necrosis factor-alpha (TNF-α), interleukin (IL)-1β, transforming growth factor-β (TGF-β)]^[18]^. Over a 30-50-year latency, these genotoxic and inflammatory pressures drive malignant transformation. Genomically, MPM is dominated by loss-of-function alterations rather than classical oncogene activation. BRCA1-associated protein 1 (BAP1) loss (~60%) disrupts chromatin regulation and DNA repair, producing an “inflamed” transcriptional phenotype with enhanced interferon signaling and greater ICI sensitivity in some studies (e.g., NCT02899299, NCT01773655)^[19]^. Cyclin-dependent kinase inhibitor 2A (CDKN2A) deletion (~50%) inactivates the retinoblastoma protein (Rb)/tumor protein p53 (p53) pathways, correlates with non-epithelioid histology and poor prognosis, and may impair antigen presentation^[20]^. Neurofibromin 2 (NF2) alterations (~40%) activate the Hippo–Yes-associated protein (YAP) axis, promoting proliferation, epithelial-mesenchymal transition (EMT), and immunosuppressive cytokines [IL-6, C-X-C motif chemokine ligand 5 (CXCL5)], along with programmed death-ligand 1 (PD-L1) and vascular endothelial growth factor (VEGF) upregulation; transcriptional enhanced associate domain (TEAD) inhibitors (e.g., IK-930, NCT05228015) are under clinical evaluation in NF2-mutant tumors. TEAD inhibition specifically addresses the Hippo pathway dysregulation discussed in Section MECHANISTIC INSIGHTS INTO THERAPEUTIC ACTION, restoring immune susceptibility in NF2-mutant tumors by modulating the immunosuppressive cytokine milieu and potentially enhancing susceptibility to ICI therapy. Sarcomatoid MPM displays a distinct immunobiological profile characterized by elevated PD-L1 expression, enhanced interferon-related transcriptional programs, and increased immune infiltration compared with epithelioid tumors. Notably, subgroup analyses from CheckMate 743 demonstrated that patients with non-epithelioid histology derived a disproportionately greater survival benefit from dual immune checkpoint blockade, suggesting that sarcomatoid tumors may represent an inflamed yet immunologically restrained subtype particularly amenable to checkpoint-based strategies. Recent multiplex immunofluorescence analyses have further elucidated the molecular basis of this heightened sensitivity. A study using an 8-marker panel revealed that sarcomatoid tumors exhibit significantly higher proportions of cells expressing cyclooxygenase-2 (COX2) (92.6% *vs.* 80.9%, *P* < 0.001), transforming growth factor-beta (TGFB) (86.3% *vs.* 65.0%, *P* < 0.001), T-cell immunoreceptor with Ig and ITIM domains (TIGIT, 91.7% *vs.* 67.4%, *P* < 0.001), and T-cell immunoglobulin and mucin domain-containing protein 3 (TIM3) (59.7% *vs.* 20.6%, *P* < 0.001) compared to epithelioid tumors. These findings provide a mechanistic rationale for the enhanced responsiveness of sarcomatoid MPM to dual checkpoint blockade and identify potential combinatorial targets for this aggressive subtype^[21,22]^.

These genomic aberrations converge with a profoundly immunosuppressive tumor microenvironment (TME). MPM lesions are enriched in M2-polarized tumor-associated macrophages (TAMs) producing IL-10, TGF-β, and VEGF, suppressing dendritic cell (DC) maturation and correlating with poor survival^[23]^. Regulatory T cells (Tregs) and myeloid-derived suppressor cells (MDSCs) expand in response to chronic inflammation, dampening cytotoxic T-cell proliferation via IL-10, TGF-β, and arginase-1–mediated L-arginine depletion - a mechanism reinforced by tumor-intrinsic argininosuccinate synthase 1 (ASS1) deficiency^[24,25]^. VEGF-driven angiogenesis and hypoxia further impair T-cell trafficking while promoting Treg/MDSC recruitment, providing the rationale for anti-angiogenic and ICI combinations validated in BEAT-meso (NCT03762018)^[25,26]^. MPM additionally expresses multiple checkpoint and inhibitory molecules [PD-L1, Galectin-9, B7 Homolog 3 (B7-H3)], while TGF-β signaling suppresses major histocompatibility complex class I (MHC-I) and facilitates fibroblast activation; such pathways contribute to resistance to programmed cell death protein 1 (PD-1)/PD-L1 blockade and underpin ongoing TGF-β–targeted trials [e.g., TGF-β2-targeting antisense oligonucleotide (OT-101) + pembrolizumab, NCT05425576]^[26-29]^.

Together, asbestos-induced oxidative stress, tumor-suppressor loss, and a structurally and functionally immunosuppressive TME form a continuous pathogenic axis that drives tumor initiation and immune escape. These intertwined mechanisms constitute the biological framework guiding current immunotherapy strategies and next-generation approaches aimed at metabolic targeting, TME reprogramming, and pathway-specific intervention^[30,31]^.

## CLINICAL DEVELOPMENT OF ICIS IN MPM

The clinical development of ICIs in MPM has evolved from early exploratory phase II studies to multiple phase III randomized controlled trials, establishing immunotherapy as a new therapeutic pillar beyond platinum–pemetrexed chemotherapy. Early evidence came from the MAPS2 trial (NCT02716272), which compared nivolumab alone *vs.* nivolumab plus ipilimumab in relapsed MPM^[32]^. The 12-week disease control rate (DCR) reached 40% and 52%, respectively, with a median OS of 11.9 and 15.9 months - demonstrating manageable safety but increased irAEs in the dual-checkpoint cohort. In contrast, the PROMISE-meso trial (NCT02991482) compared pembrolizumab with investigator’s-choice chemotherapy and reported a higher ORR (22% *vs.* 6%) but no significant OS or PFS benefit, underscoring the limitation of PD-1 monotherapy^[33,34]^.

Definitive phase III evidence came from the CheckMate 743 trial, which compared nivolumab plus ipilimumab against platinum–pemetrexed in the first-line setting. The dual-immunotherapy regimen achieved a median OS of 18.1 *vs.* 14.1 months (HR ≈ 0.73), with durable 3-year survival rates of 23.2% *vs.* 15.4%^[35]^. Notably, the survival advantage was most pronounced in non-epithelioid histology (OS 18.1 *vs.* 8.8 months, HR ≈ 0.46), reflecting the biologic heterogeneity of immune responsiveness in MPM. These findings led to the U.S. Food and Drug Administration (FDA) approval of nivolumab–ipilimumab as first-line therapy in 2020, marking a paradigm shift in disease management^[36-38]^.

More recently, combination chemoimmunotherapy has emerged as a potential refinement. The KEYNOTE-483 trial (IND227) evaluated pembrolizumab with platinum–pemetrexed *vs.* chemotherapy alone and demonstrated a median OS of 17.3 *vs.* 16.1 months (HR = 0.79), supporting regulatory recognition in 2024^[8]^. The ongoing DREAM3R (NCT04334759) and BEAT-meso trials (NCT03762018) are further investigating immunotherapy integrated with chemotherapy and anti-angiogenic agents^[39]^. Despite these advances, response rates remain modest, long-term survival is limited to a subset of patients, and immune-related toxicities present new management challenges^[40]^. Collectively, these data highlight that while ICIs have redefined the therapeutic landscape of MPM, overcoming primary and acquired resistance remains the next frontier^[41,42]^.

## MECHANISTIC INSIGHTS INTO THERAPEUTIC ACTION

The limited efficacy and heterogeneous clinical response to ICIs in MPM reflect a complex interplay of immune, stromal, metabolic, and genomic mechanisms that collectively drive both primary refractoriness and acquired resistance during treatment. A defining pathological feature of MPM is its chronically inflamed yet immunologically “restricted” TME, where effector T-cell recruitment, activation, and long-term persistence are impeded by multiple layers of suppressive signaling^[43]^. At the cellular level, enrichment of Tregs constitutes a major barrier to antitumor immunity^[44,45]^. These cells accumulate in pleural tumor nodules and effusions, maintained by continuous exposure to IL-6, TGF-β, and tumor-derived prostaglandins^[46]^. Once present, Tregs inhibit DC maturation, outcompete CD8^+^ T cells for IL-2, and deliver direct suppressive signals via CTLA-4–mediated trans-endocytosis of CD80/86 from antigen-presenting cells. This constellation of effects results in attenuated priming and impaired expansion of tumor-reactive T-cell clones^[47-50]^.

MDSCs further reinforce this tolerance landscape. Driven by persistent asbestos-induced inflammation, high mobility group box 1 protein (HMGB1) release, and tumor-secreted granulocyte-macrophage colony-stimulating factor (GM-CSF) and IL-8, MDSCs accumulate in both systemic circulation and the pleural cavity^[50]^. Through high-level expression of arginase-1, inducible nitric oxide synthase (iNOS), and ROS–generating enzymes, MDSCs inhibit T-cell receptor (TCR) ζ-chain expression, curtail antigen-specific proliferation, and restrict T-cell infiltration via chemokine remodeling^[51]^. Importantly, this immunosuppressive program converges with a core metabolic vulnerability of MPM: deficiency of ASS1. Loss of ASS1 - especially prevalent in non-epithelioid tumors - renders MPM highly dependent on exogenous arginine. As MDSCs consume arginine at a high rate, effector lymphocytes are metabolically starved, creating a competitive disadvantage that profoundly limits their activation threshold and cytotoxic capacity. These processes act in concert to establish a baseline state of ICI resistance even before treatment begins^[52-55]^.

In addition to cellular and metabolic obstacles, intrinsic genomic features shape ICI responsiveness. MPM typically carries a low tumor mutational burden (TMB), limiting neoantigen availability and reducing the probability of pre-existing T-cell clones capable of recognizing tumor antigens^[56]^. Loss-of-function alterations in BAP1, NF2, and components of the Hippo–YAP signaling pathway modulate interferon signaling, antigen presentation, and cytokine release. While some BAP1-deficient tumors exhibit increased immune infiltration, a significant proportion display impaired type I interferon responses and reduced MHC class I expression, enabling immune evasion and promoting the emergence of antigen-loss variants during ICI therapy^[57,58]^. NF2 mutation, ASS1 deficiency, and BAP1 loss should not be viewed as isolated genomic events but as components of an interconnected metabolic–immune circuitry. NF2 inactivation drives Hippo pathway dysregulation and mammalian target of rapamycin (mTOR) activation, thereby increasing anabolic demand and nutrient dependence. In the setting of ASS1 deficiency, enforced arginine auxotrophy imposes metabolic rigidity, coupling proliferative signaling to nutrient vulnerability. This convergence may simultaneously sensitize tumors to arginine deprivation and intensify metabolic competition within the TME, with potential consequences for T-cell fitness and immune responsiveness. By contrast, BAP1 loss confers context-dependent immunologic effects. While frequently associated with interferon signaling, increased CD8^+^ infiltration, and elevated PD-L1 expression, BAP1 deficiency may also facilitate compensatory immune-evasive pathways and metabolic rewiring. Its impact on immune checkpoint blockade is therefore shaped by histologic context and co-occurring genomic alterations, underscoring the need to interpret BAP1 loss within a broader immunogenomic framework rather than as a standalone predictive marker. These interconnected vulnerabilities provide a rationale for mechanism-guided combinatorial strategies in MPM^[59-63]^.

The stromal architecture of MPM further compounds therapeutic resistance. Dense fibrous stroma, abundant cancer-associated fibroblasts (CAFs), and dysfunctional neovasculature driven by VEGF impede lymphocyte trafficking and generate hypoxic, lactate-rich niches that destabilize T-cell mitochondrial metabolism^[64]^. VEGF-mediated inhibition of DC maturation also contributes to insufficient antigen priming, reinforcing a feedback loop of ineffective T-cell–mediated antitumor surveillance. These stromal barriers are particularly relevant in patients receiving ICIs, where the absence of adequate lymphocyte infiltration precludes the establishment of sustained antitumor immunity^[65-68]^.

Cytokine-mediated mechanisms also contribute to treatment failure. High baseline levels of TGF-β, IL-10, and IL-1β suppress T-cell recruitment and effector differentiation, promote EMT, and remodel fibroblast and macrophage phenotypes toward immunosuppressive states^[69,70]^. During ICI therapy, compensatory upregulation of alternative checkpoints, such as T-cell immunoglobulin and mucin-domain containing-3 (TIM-3), lymphocyte-activation gene-3 (LAG-3), TIGIT, and V-domain immunoglobulin (Ig) suppressor of T cell activation (VISTA), further contributes to T-cell exhaustion and loss of cytotoxic function. These adaptive pathways represent hallmark signatures of acquired resistance across multiple clinical cohorts^[71-75]^.

Collectively, these intertwined cellular, stromal, metabolic, genomic, and cytokine-driven factors establish a multilayered resistance architecture unique to MPM. Understanding this complex ecosystem is essential for guiding rational combination strategies - particularly those integrating ICIs with metabolic modulation, anti-angiogenic agents, TGF-β inhibition, cellular immunotherapies, and genetically informed targeted agents. Overcoming resistance in MPM requires simultaneous disruption of multiple suppressive circuits rather than reliance on any single therapeutic axis, reinforcing the need for precision, multimodal immuno-oncologic approaches^[76-79]^.

**Figure 1:**
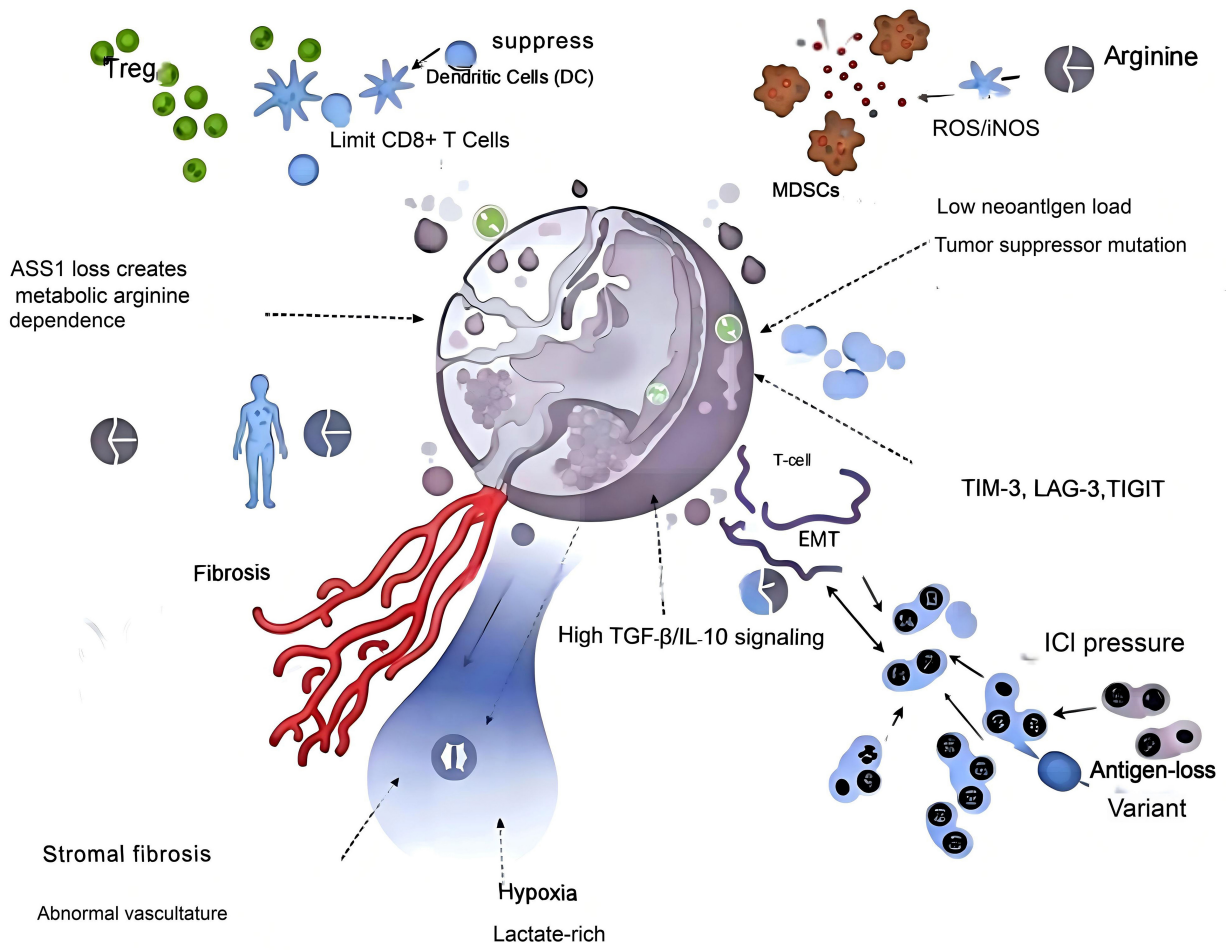
Key drivers of T cell–mediated immune resistance in MPM. MPM: Malignant pleural mesothelioma; Treg: regulatory T cell; DC: dendritic cell; CD8^+^ T cell: cytotoxic T lymphocyte; MDSCs: myeloid-derived suppressor cells; ROS: reactive oxygen species; iNOS: inducible nitric oxide synthase; ASS1: argininosuccinate synthase 1; TGF-β: transforming growth factor beta; IL-10: interleukin-10; EMT: epithelial-mesenchymal transition; TIM-3: T-cell immunoglobulin and mucin-domain containing-3; LAG-3: lymphocyte-activation gene-3; TIGIT: T-cell immunoreceptor with Ig and ITIM domains; ICI: immune checkpoint inhibitor.

As summarized in Figure 1, these convergent pathways - including ASS1-mediated metabolic arginine dependency, Treg and MDSC accumulation, fibroblast-driven stromal stiffening, hypoxia, and alternative immune checkpoint upregulation - form a multilayered suppressive network that restrains cytotoxic T-cell infiltration and effector function.

## STRATEGIES TO OVERCOME IMMUNOTHERAPY RESISTANCE IN MPM

**Figure 2:**
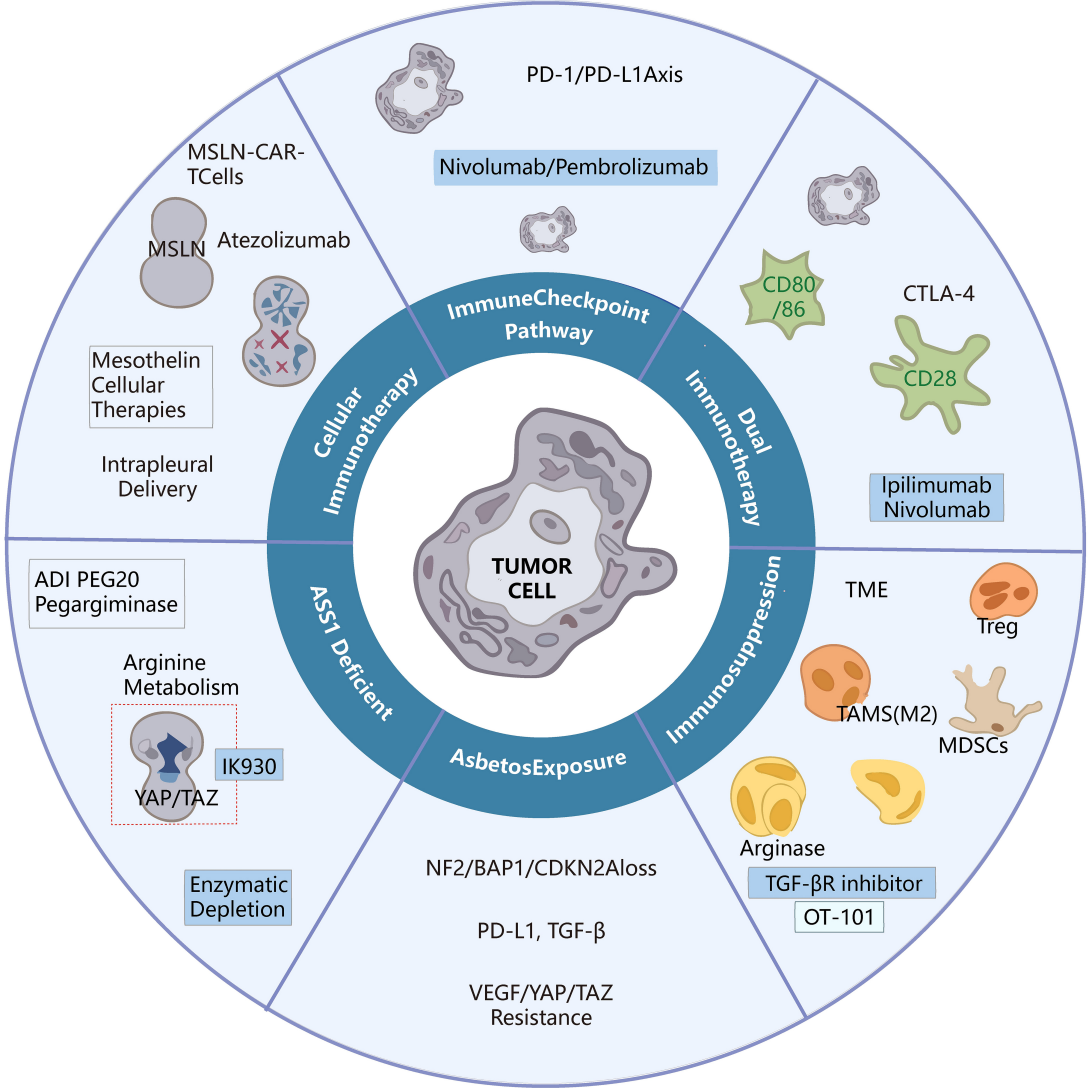
Strategies to overcome immunotherapy resistance in MPM. MPM: Malignant pleural mesothelioma; MSLN: mesothelin; CAR-T: chimeric antigen receptor T cell; Atezolizumab: anti-PD-L1 monoclonal antibody; PD-1: programmed cell death protein 1; Nivolumab: anti-PD-1 monoclonal antibody; Pembrolizumab: anti-PD-1 monoclonal antibody; CTLA-4: cytotoxic T-lymphocyte-associated protein 4; Ipilimumab: anti-CTLA-4 monoclonal antibody; TME: tumor microenvironment; Treg: regulatory T cell; TAMs: tumor-associated macrophages; MDSCs: myeloid-derived suppressor cells; TGF-βR: transforming growth factor-β receptor; OT-101: TGF-β2-targeting antisense oligonucleotide; NF2: neurofibromin 2; BAP1: BRCA1-associated protein 1; CDKN2A: cyclin-dependent kinase inhibitor 2A; PD-L1: programmed death-ligand 1; TGF-β: transforming growth factor-β; VEGF: vascular endothelial growth factor; YAP: Yes-associated protein; TAZ: transcriptional coactivator with PDZ-binding motif; ADI-PEG20: PEGylated arginine deiminase; Pegargininase: synonym for ADI-PEG20.

The multifaceted resistance mechanisms delineated in this chapter - ranging from cellular suppressors (Tregs, MDSCs) and metabolic constraints (ASS1 deficiency) to genomic alterations (BAP1, NF2, CDKN2A) and stromal barriers (CAFs, VEGF-driven angiogenesis) - provide a rational framework for designing intervention strategies. Rather than targeting these obstacles in isolation, effective therapeutic combinations must simultaneously disrupt multiple layers of this suppressive network^[80-83]^. This section systematically maps each major resistance mechanism to its corresponding counter-strategy, establishing a mechanistic closed loop between the vulnerabilities characterized in Section MECHANISTIC INSIGHTS INTO THERAPEUTIC ACTION and their therapeutic targeting in Section STRATEGIES TO OVERCOME IMMUNOTHERAPY RESISTANCE IN MPM. Given the multifactorial nature of primary and acquired resistance in MPM, rational therapeutic strategies have increasingly focused on reprogramming the immunosuppressive TME, restoring antigen presentation, enhancing T-cell infiltration, and targeting tumor-specific metabolic and signaling dependencies. To systematically illustrate the key therapeutic directions that have emerged, Table 1 summarizes the representative clinical trials conducted from 2020-2025 exploring different approaches to overcoming immunotherapy resistance in MPM. This chapter provides a comprehensive synthesis of the most promising strategies designed to dismantle these resistance mechanisms, with the ultimate goal of restoring and sustaining effective anti-tumor immunity in MPM^[32,84-87]^. These strategies include anti-angiogenic approaches, chemo-immunotherapy combinations, metabolic modulation (e.g., arginine deprivation), TGF-β blockade, chimeric antigen receptor T cell (CAR-T)-based cellular immunotherapy, oncolytic viral therapy, dendritic-cell vaccination, and epigenetic/Hippo-pathway targeting. Figure 2 provides an illustrated overview of the immunosuppressive network in MPM and how each intervention point disrupts immune escape mechanisms.

**Table 1:** Representative trials addressing strategies to overcome resistance to immunotherapy in MPM (2020-2025)

**Representative trials (NCT/year)**	**Phase/*n***	**Strategy**	**Key outcomes**	**Mechanistic rationale**	**Ref.**
BEAT-meso (NCT03762018, 2024)	Phase III / *n* = 400	Atezolizumab + Bevacizumab + Carboplatin + Pemetrexed *vs.* Bevacizumab + chemotherapy	ORR: 55% *vs.* 49% (NS); PFS: 9.2 *vs.* 7.6 mon (HR = 0.72, *P* = 0.0021); OS: 20.5 *vs.* 18.1 mon (HR = 0.84, NS); non-epithelioid subgroup OS HR = 0.51	VEGF blockade normalizes vasculature, decreases Tregs/M2-TAMs, improves T-cell infiltration	[9,107,108,110]
KEYNOTE-483 (NCT02784171, 2023-2024); PrE0505 (NCT02899195, 2022)	Phase III / *n* = 539; Phase II / *n* = 55	Phase III: Pembrolizumab + Platinum/Pemetrexed *vs.* chemotherapy Phase II: Durvalumab + Platinum/Pemetrexed	Phase III: ORR: 52% *vs.* 29%; PFS: 7.1 *vs.* 7.1 mon (NS); OS: 17.3 *vs.* 16.1 mon Phase II: ORR: 56% (BICR); DCR: 92%; PFS: 6.7 mon; OS: 20.4 mon	Cytotoxic therapy induces ICD and DAMP release to prime DC-T cell axis	[40,89,91,96]
ATOMIC-Meso (NCT02709512, 2024)	Phase II/III / *n* = 249	Pegargiminase + chemotherapy *vs.* chemotherapy	PFS: 6.1 *vs.* 5.6 mon (HR = 0.65); OS: 9.3 *vs.* 7.7 mon (HR = 0.71)	ASS1-deficient tumors depend on extracellular arginine; deprivation enhances T-cell function	[25,36,80,86]
OT-101 + Pembrolizumab (NCT05425576, ongoing)	Phase II / ongoing	TGF-β inhibition + ICI	ORR: not reported PFS: not reported OS: not reported Safety: preliminary data suggest good tolerability	TGF-β drives Treg induction and fibrosis; blockade restores cytotoxic function	[26,27]
NCT02414269 (2022, Nat Med); NCT04577326 (ongoing)	Phase I/II / *n* = 21 + 36	Cellular immunotherapy (CAR-T)	ORR: 12.5% (2/16 PR); DCR: 68.8%; PFS/OS: not reported	Local delivery of MSLN-targeted CAR-T enhances tumor infiltration; PD-1 inhibition prevents exhaustion	[113,118,126]
Gavo-cel (NCT03585764, 2023)	Phase I / *n* = 39	TRuC-T therapy	ORR: 21% (BICR) / 26% (investigator); PFS: 5.6 mon; OS: 11.2 mon	TCR-fusion construct enhances immune synapse formation and persistence	[126]
ONCOS-102 (NCT02879669, 2020); INFINITE (NCT03710876, 2025)	Phase I / *n* = 12; Phase III / *n* = 280	Oncolytic virus therapy	ONCOS-102: ORR: 30%; DCR: 90% PFS: 9.8 mon; OS: 25.0 *vs.* 13.5 mon; Safety: well tolerated, no DLT INFINITE: ORR: ~25% (based on early Phase II) DCR: ~87.5% PFS: not reported OS: not reported Safety: manageable (early data)	Viral lysis releases tumor antigens, converts “cold” to “hot” TME	[144,145]
DENIM (NCT03610360, 2025); MESOVAX (NCT03546426, 2023)	Phase III / *n* ≈ 150; Phase I/II / *n* = 15	DENIM: DC vaccines MESOVAX: autologous DCs + tumor lysate	DENIM: PFS: 4.1 mon; OS: 11.9 mon MESOVAX: ORR: 56% (BICR); PFS: 6.7 mon; OS: 20.4 mon	DC vaccines enhance antigen presentation and ICI synergy	[40,83,97,99]
IK-930 (NCT05228015, ongoing)	Phase I / *n* ≈ 40	Oral TEAD inhibitor	ORR/PFS/OS: not reported (early-phase study)	EZH2 or TEAD inhibition restores antigenicity and interferon signaling	[62,63]

MPM: Malignant pleural mesothelioma; ORR: objective response rate; NS: not significant; PFS: progression-free survival; HR: hazard ratio; OS: overall survival; VEGF: vascular endothelial growth factor; Tregs: regulatory T cells; TAMs: tumor-associated macrophages; BICR: blinded independent central review; DCR: disease control rate; ICD: immunogenic cell death; DAMP: damage-associated molecular pattern; DC: dendritic cell; ASS1: argininosuccinate synthase 1; TGF-β: transforming growth factor beta; ICI: immune checkpoint inhibitor; CAR-T: chimeric antigen receptor T cell; PR: partial response; MSLN: mesothelin; PD-1: programmed cell death protein 1; TRuC-T: TCR fusion construct T cell; DLT: dose-limiting toxicity; TME: tumor microenvironment; TEAD: transcriptional enhanced associate domain; EZH2: enhancer of zeste homolog 2.

### Chemo-immunotherapy to potentiate immunogenic cell death

The combination of conventional cytotoxic chemotherapy with ICIs may seem counterintuitive, given the historical view of chemotherapy as being immunosuppressive. However, a paradigm shift has occurred with the understanding that certain chemotherapeutic agents can induce immunogenic cell death (ICD) platinum-based drugs and pemetrexed, the backbone of MPM chemotherapy, are potent inducers of ICD^[88,89]^. Representative clinical trials evaluating this strategy are summarized in Table 2. This specialized form of apoptosis is characterized by the exposure and release of damage-associated molecular patterns (DAMPs), including the translocation of calreticulin to the cell surface, the extracellular release of ATP, and the passive secretion of HMGB1^[90]^. These “danger signals” act as potent adjuvants: they drive the maturation and antigen-presenting function of DCs, enhance the cross-presentation of tumor antigens to naïve T cells, and ultimately amplify the priming and clonal expansion of tumor-specific CD8^+^ T cells. This process effectively turns chemotherapy into an *in situ* vaccine, thereby counteracting one of the principal causes of ICI failure: inadequate initial T-cell activation and priming^[91-94]^.

**Table 2:** ICIs with combination therapies - updated table (2020-2025)

**Representative trials (NCT/year)**	**Phase**	**Patients (*n*)**	**Agent/strategy**	**Key outcomes**	**Target/pathway**	**Ref.**
MAPS2 (NCT02716272, 2019. Updated 2023)	Phase II	125	Nivolumab ± Ipilimumab	ORR: 28% (combination); PFS: 3.0 mon; OS: 15.9 mon (combo) *vs.* 11.9 mon (monotherapy)	PD-1/CTLA-4 dual blockade - T-cell activation	[34]
PROMISE-meso (NCT02991482, 2020)	Phase III	144	Pembrolizumab *vs.* single-agent chemo	ORR: 22% *vs.* 6% (*P* = 0.004); PFS: 2.5 *vs.* 3.4 mon (HR = 1.06; *P* = 0.76); OS: 10.7 *vs.* 11.7 mon (HR = 1.12; 95%CI 0.74-1.69; *P* = 0.59)	PD-1 blockade - immune checkpoint inhibition	[105]
CheckMate 743 (2021-2023)	Phase III	605	Nivolumab + Ipilimumab *vs.* platinum-pemetrexed	ORR: 40% *vs.* 44%; median PFS: not consistently reported; 3-year PFS rate: ~14% *vs.* ~1%; OS: 18.1 *vs.* 14.1 mon (HR = 0.73); 3-year OS rate: ~23% *vs.* ~15%	Dual PD-1/CTLA-4 pathway - durable immune memory	[171]
KEYNOTE-483 (FDA-approved 2024)	Phase III	Approx.440	Pembrolizumab + platinum-pemetrexed *vs.* chemo	ORR: 52% *vs.* 29%; PFS: 7.1 *vs.* 7.1 mon; OS: 17.3 *vs.* 16.1 mon	PD-1 inhibition + cytotoxic synergy	[96]
BEAT-meso (NCT03762018, 2025)	Phase III	400	Atezolizumab + Bevacizumab + chemo *vs.* Bevacizumab + chemo	ORR: 55% *vs.* 49%; PFS: 9.8 *vs.* 7.6 mon; OS: 25.0 *vs.* 13.5 mon	PD-L1 blockade + VEGF inhibition - immune-angiogenic synergy	[9,107,108]
OT-101 + Pembrolizumab (NCT05425576, Ongoing)	Phase II	-	TGF-β2 antisense (OT-101) + PD-1 inhibitor	ORR: not reported PFS: not reported OS: not reported Safety: preliminary data suggest good tolerability	TGF-β pathway blockade - reversing immune suppression	[26]
Avelumab + SBRT (NCT03399552, 2023)	Phase I/II	Small	PD-L1 inhibitor + stereotactic body radiation therapy	ORR/PFS/OS: not reported (safety study)	PD-L1 blockade + radiation-induced antigen release	ClinicalTrials.gov
DREAM/DREAM3R (NCT04334759, ongoing)	Phase II/III	-	Durvalumab + platinum-pemetrexed (± maintenance)	ORR/PFS/OS: not reported	PD-L1 blockade + chemo - chemo-immune synergy	[98]
NEMO (NCT02863055, ongoing)	Phase III	116	Nintedanib + platinum/pemetrexed	ORR/PFS/OS: not reported	Multi-target angiokinase inhibition (VEGFR, PDGFR, FGFR)	[108]
ATOMIC-Meso (NCT02709512, 2024)	Phase II/III	249	ADI-PEG20 + chemo *vs.* placebo + chemo	Median OS: 9.3 *vs.* 7.7 mon (HR = 0.71); Median PFS: 6.2 *vs.* 5.6 mon	Arginine deprivation - metabolic immunomodulation	[36]
IK-930 (NCT05228015, ongoing)	Phase I	-	Oral TEAD inhibitor (Hippo pathway)	ORR/PFS/OS: not reported (early-phase study)	TEAD/YAP-TAZ - tumor suppressor restoration	[62,63]
PENINSULA (NCT05412615, 2026 ongoing)	Phase II	Planned *n* = 25	Pembrolizumab + Lenvatinib + Platinum/Pemetrexed	ORR/PFS/OS: not reported	PD-1 blockade + VEGFR inhibition + cytotoxic chemotherapy	ClinicalTrials.gov
MesoNet	Real-world retrospective	*n* = 135	First-line nivolumab + ipilimumab (real-world validation)	OS (overall): 13.1 mon; OS (CM-743 eligible): 15.5 mon; Non-epithelioid OS: 16.7 mon	Dual PD-1/CTLA-4 blockade (real-world confirmation)	[206]

ICIs: Immune checkpoint inhibitors; ORR: objective response rate; PFS: progression-free survival; OS: overall survival; PD-1: programmed cell death protein 1; CTLA-4: cytotoxic T-lymphocyte-associated protein 4; HR: hazard ratio; CI: confidence interval; VEGF: vascular endothelial growth factor; TGF-β2: transforming growth factor beta 2; SBRT: stereotactic body radiation therapy; PD-L1: programmed death-ligand 1; VEGFR: vascular endothelial growth factor receptor; PDGFR: platelet-derived growth factor receptor; FGFR: fibroblast growth factor receptor; ADI-PEG20: arginine deiminase PEGylated 20; TEAD: transcriptional enhanced associate domain; YAP: Yes-associated protein; TAZ: transcriptional coactivator with PDZ-binding motif.

The randomized, global phase III KEYNOTE-483 (also known as IND227; NCT02784171) trial provides robust clinical validation for this strategy. This pivotal study, which formed the basis for the 2024 FDA approval of the regimen, evaluated pembrolizumab (anti-PD-1) in combination with platinum-pemetrexed chemotherapy *vs.* chemotherapy alone in 539 patients with previously untreated, unresectable MPM. The combination therapy achieved a modest but clinically meaningful improvement in the primary endpoint of OS, with a median OS of 17.3 months compared to 16.1 months in the chemotherapy-alone arm (HR = 0.79). Consistent with other immunotherapy trials, a more pronounced benefit was observed in the non-epithelioid subgroup. These results validate chemo-immunotherapy as a viable and effective approach to overcoming poor immune priming^[95-98]^.

Further compelling support comes from the single-arm phase II PrE0505 trial (NCT02899195), in which durvalumab (anti-PD-L1) was combined with cisplatin and pemetrexed. This regimen yielded an impressive median OS of 20.4 months - a result that compares favorably with, and in some selected patient subsets may even exceed, outcomes observed with dual ICI regimens. Collectively, the evidence from KEYNOTE-483 and PrE0505 underscores a fundamental principle: that enhancing tumor antigen release and T-cell priming through chemotherapy can effectively mitigate the primary resistance to PD-1/PD-L1 blockade that is observed in a significant number of MPM patients^[99-102]^.

In patients with unresectable MPM who progress after first-line nivolumab plus ipilimumab, effective second-line options remain limited. The phase 2 PEMMELA study (cohort 2) evaluated pembrolizumab plus lenvatinib in this setting and reported an investigator-assessed ORR of 60% (12/20; 95%CI, 36%-81%), with a median PFS of 6.9 months and median OS of 14.1 months. Although grade 3-4 treatment-related adverse events occurred in 70% of patients, these results suggest that immunotherapy combined with targeted therapy may represent a promising strategy to overcome resistance to prior dual immune checkpoint blockade^[103-105]^.

### Angiogenesis inhibition to reprogram the TME and restore immunotherapy sensitivity

Pathological angiogenesis is a cornerstone of the immunosuppressive TME in MPM. VEGF, often overexpressed in MPM, does more than simply stimulate the formation of disorganized, leaky, and dysfunctional blood vessels that impede the efficient trafficking of cytotoxic T cells into the tumor core. It also acts as a direct mediator of immune suppression. VEGF signaling inhibits T-cell proliferation and cytotoxic function, impairs the maturation and antigen-presenting capacity of DCs, and actively promotes the recruitment and expansion of Tregs and M2-polarized TAMs. Thus, VEGF blockade represents a dual-pronged therapeutic strategy: it promotes “vascular normalization”, which improves perfusion and T-cell infiltration, while simultaneously dismantling a key immunosuppressive axis within the TME^[106-108]^.

The phase III BEAT-meso trial (NCT03762018) stands as the most definitive clinical evaluation of this approach to date. This randomized study compared the efficacy of atezolizumab (anti-PD-L1) plus bevacizumab (anti-VEGF) and platinum-pemetrexed chemotherapy [the atezolizumab, bevacizumab, and platinum-pemetrexed chemotherapy (ABC) regimen] against bevacizumab plus chemotherapy alone [the bevacizumab plus platinum-pemetrexed chemotherapy (BC) regimen] in 400 previously untreated, unresectable MPM patients. Results, published in *Annals of Oncology* in 2025, demonstrated a statistically significant and clinically meaningful improvement in the co-primary endpoint of PFS, with a median PFS of 9.2 months in the ABC arm *vs.* 7.6 months in the BC arm (HR = 0.72). Although the other co-primary endpoint, OS, did not cross the statistical significance boundary (median OS 20.5 *vs.* 18.1 months; *P* = 0.14), a critical prespecified subgroup analysis revealed a pronounced OS benefit in patients with non-epithelioid histology (HR = 0.51). This finding reinforces the concept of histology-specific biology. It suggests that the more aggressive, chemoresistant non-epithelioid subtypes, which are often highly angiogenic, may derive exceptional benefit from combined angiogenesis and immune checkpoint inhibition. Mechanistically, bevacizumab-mediated VEGF inhibition has been shown in translational studies to reduce Treg accumulation, inhibit M2 TAM polarization, and facilitate CD8^+^ T-cell entry into tumor islets, providing a sound biological rationale for this combination. By inhibiting VEGF signaling, this approach not only reverses the immunosuppressive environment driven by aberrant angiogenesis but also restores dysfunctional vascular architecture described in Section MECHANISTIC INSIGHTS INTO THERAPEUTIC ACTION. This dual action provides a mechanistic foundation for its synergistic effect with ICIs, which is particularly pronounced in the highly angiogenic and inherently resistant non-epithelioid subtype. Ongoing studies, such as those evaluating multikinase inhibitors with potent anti-angiogenic properties [e.g., nintedanib in the NEMO trial (NCT02863055, Ongoing)], may help refine our understanding of vascular modulation and reinforce its centrality in reconditioning the TME to support sustained ICI efficacy^[109-111]^.

### Metabolic reprogramming as an immune-modulatory therapeutic avenue in MPM

Metabolic competition within the TME constitutes a critical and often overlooked barrier to effective immunotherapy. A hallmark of MPM, particularly the non-epithelioid subtype, is the frequent epigenetic silencing and loss of ASS1, a key enzyme in the de novo synthesis of arginine. This loss renders cancer cells auxotrophic for extracellular arginine, creating a profound metabolic dependency. This dependency has two major implications for ICI resistance. First, it instigates a fierce nutrient competition between rapidly dividing tumor cells and infiltrating T cells, for which arginine is an essential nutrient required for TCR signaling, proliferation, and survival. Second, this competition is exacerbated by elevated arginase-1 activity from MDSCs within the TME, which further depletes local arginine pools, leading to T-cell dysfunction, cell cycle arrest, and functional paralysis^[112]^.

The phase II/III ATOMIC-Meso trial (NCT02709512) was designed to exploit this very vulnerability. The study evaluated the efficacy of pegargiminase [PEGylated Arginine Deiminase (ADI-PEG20)], a microbially derived arginine-degrading enzyme, in combination with first-line platinum–pemetrexed chemotherapy in 249 patients with ASS1-deficient MPM. As reported in *JAMA Oncology* in 2024, the combination demonstrated statistically significant improvements in both OS (median OS 9.3 *vs.* 7.7 months; HR = 0.71) and PFS (median PFS 6.2 *vs.* 5.6 months). While the primary anti-tumor effect of ADI-PEG20 is direct cytotoxicity against ASS1-deficient cancer cells by inducing arginine starvation, an important indirect immunomodulatory effect is now appreciated. By systemically depleting arginine, ADI-PEG20 may reduce the tumor’s nutrient consumption and potentially alter MDSC function, thereby replenishing bioavailable arginine for T-cell activation and attenuating immunosuppressive enzymatic activity in the TME. This may ultimately promote the restoration of T-cell effector functions^[36]^. This approach directly targets the metabolic vulnerability of ASS1-deficient tumors discussed in Section MECHANISTIC INSIGHTS INTO THERAPEUTIC ACTION, alleviating nutrient competition between cancer cells and tumor-infiltrating lymphocytes (TILs) and providing a mechanistic rationale for combining ADI-PEG20 with ICIs.

The success of ATOMIC-Meso has unequivocally established metabolic immunomodulation as a promising and novel therapeutic avenue in MPM. It suggests that ASS1 status may serve as a potential predictive biomarker for patient stratification, although further prospective validation is warranted.

### Cellular immunotherapy to reinvigorate effector function

Adoptive cellular immunotherapy has emerged as one of the most promising strategies to counteract primary and acquired resistance to ICIs in MPM. Unlike ICIs, which rely on the presence of pre-existing functional T cells, adoptive cell therapies directly introduce large numbers of tumor-specific effector lymphocytes capable of overcoming T-cell exclusion, exhaustion, and suppressive stromal barriers. The pleural cavity offers a unique anatomic advantage for regional delivery, allowing high local concentrations of engineered cells while minimizing systemic toxicities typically associated with CAR-T therapies^[36,113]^.

**Figure 3:**
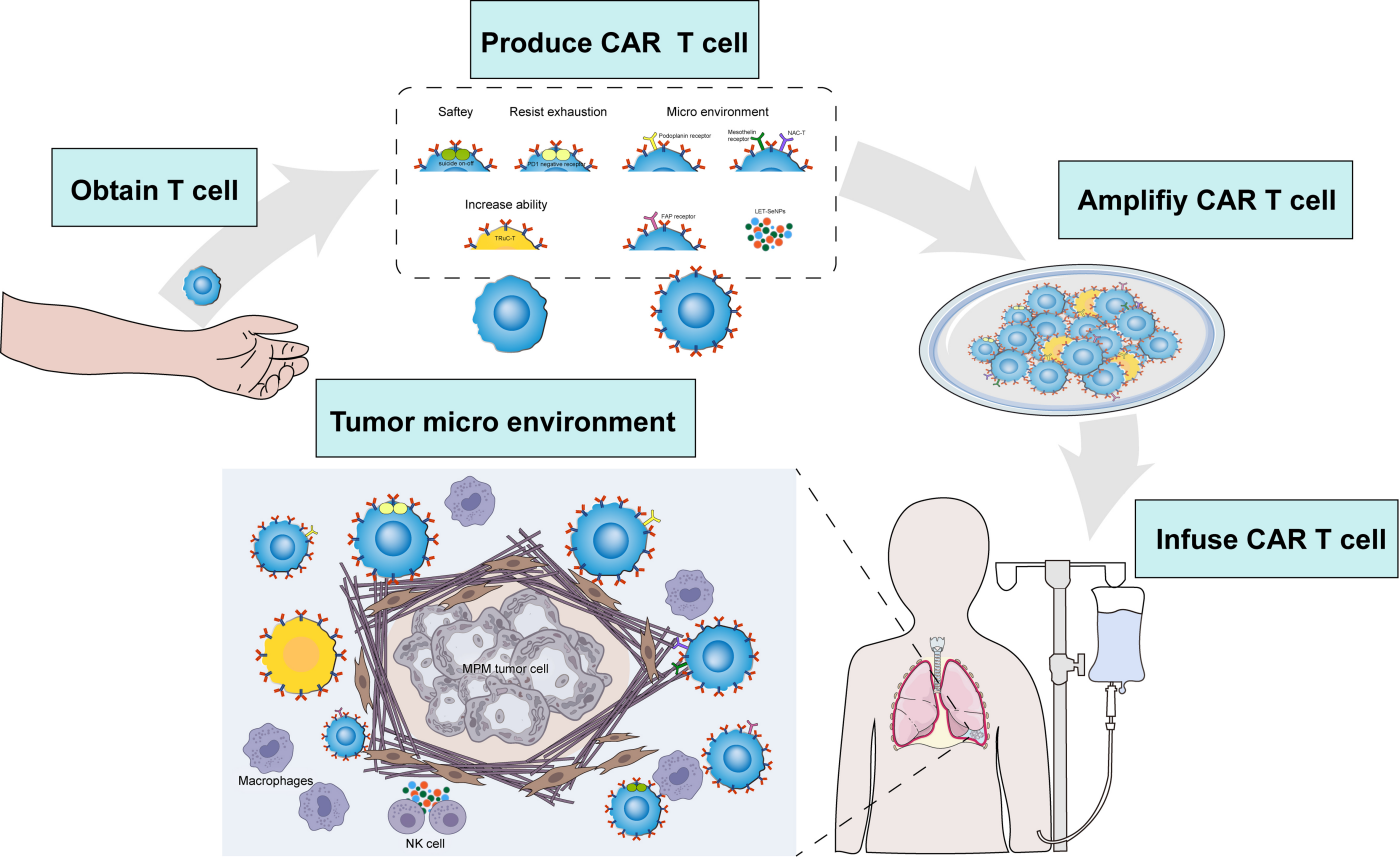
Cellular immunotherapy for MPM. MPM: Malignant pleural mesothelioma; CAR-T: chimeric antigen receptor T cells; PD1: programmed cell death protein 1; NAC-T: nanobody-armored CAR-T cells; TRuC-T: T-cell receptor fusion construct; FAP: fibroblast activation protein; LET-SeNPs: lentinan-functionalized selenium nanoparticles; NK: natural killer.

An overview of key efforts in cellular immunotherapy for MPM is summarized in Tables 3 and 4 and Figure 3.

**Table 3:** CAR-T (regional with engineering strategies)

**Representative trials (NCT/year)**	**Phase**	**Patients (*n*)**	**Agent/strategy**	**Key outcomes**	**Target/pathway**	**Ref.**
NCT02414269 (MSKCC, 2021 ASCO; 2022 Nat Med)	Phase I/II	*n* = 21 (19 MPM)	iCasp9 M28z CAR-T (anti-MSLN) + Pembrolizumab	ORR: 12.5% (2/16 PR); DCR: 68.8%; PFS/OS: not reported	MSLN CAR-T + PD-1 blockade	[113]
NCT03054298 (M5 CAR, 2022-2023 ASCO/SITC)	Phase I	Planned *n* = 18	Fully humanized MSLN CAR-T	ORR: not reported; PFS: not reported; OS: not reported	MSLN (humanized scFv, low immunogenicity)	[115-117]
NCT04577326 (ATA2271, 2023 ASCO)	Phase I	Planned *n* = 36 (ongoing)	PD-1–blocked MSLN CAR-T (intrapleural)	ORR: not reported; PFS: not reported; OS: 23.9 months (reported at ASCO 2023); 1-year OS: 83%	MSLN + PD-1–resistant CAR-T	[118] ClinicalTrials.gov
NCT03585764 (gavo-cel/TRuC-T, 2022 ASCO; 2023 Clin Cancer Res)	Phase I	*n* = 39	TRuC-T therapy	ORR: 21% (BICR) / 26% (investigator); PFS: 5.6 mon; OS: 11.2 mon	MSLN-targeted TRuC platform	[126]
NCT01722149 (FAP CAR-T, 2014 Sci Transl Med)	Phase I	*n* = 3	CAR-T targeting FAP	ORR: not reported; PFS: not reported; OS: not reported; No CRS observed	FAP (stromal target)	[119,120]
NCT03618381 (PDPN CAR-T, expected 2025)	Phase I	Planned *n* = 18 (ongoing)	CAR-T targeting PDPN	ORR: not reported; PFS: not reported; OS: not reported	PDPN	[121] ClinicalTrials.gov
FIH study (2023)	Phase I	*n* = 11	NAC-T (MSLN CAR-T armored with anti-PD-1 nanobody)	ORR: 63.6%; DCR: 100%; OS: 25.6 months; PFS: not reported; No DLT observed	MSLN/local PD-1 blockade	[127]
Published preclinical study (2025)	Preclinical	-	LET-SeNPs	Restored NK cytotoxicity, upregulated NKG2D expression, and inhibited invasion in spheroid and organoid models	TrxR1–pSTAT3/NKG2D axis	[128]

CAR-T: Chimeric antigen receptor T cell; MPM: malignant pleural mesothelioma; MSLN: mesothelin; iCasp9: inducible caspase 9; ORR: objective response rate; PR: partial response; DCR: disease control rate; PFS: progression-free survival; OS: overall survival; PD-1: programmed cell death protein 1; scFv: single-chain variable fragment; TRuC-T: TCR fusion construct T cell; BICR: blinded independent central review; FAP: fibroblast activation protein; CRS: cytokine release syndrome; PDPN: podoplanin; DLT: dose-limiting toxicity; LET-SeNPs: lentinan-functionalized selenium nanoparticles; NK: natural killer; NKG2D: natural killer group 2, member D; TrxR1-pSTAT3: thioredoxin reductase 1–phosphorylated signal transducer and activator of transcription 3.

**Table 4:** Dendritic-cell vaccines with oncolytic/viral-based cytokine delivery

**Representative trials (NCT/year)**	**Phase**	**Patients (*n*)**	**Agent/strategy**	**Key outcomes**	**Target/pathway**	**Ref.**
Autologous DC vaccines (Multiple Trials, 2018-2021)	Phase II (various)	> 100 (across studies)	Autologous DCs loaded with tumor lysate or defined antigens ± ICI	mOS: 19-27 months; 2-year OS ≈ 50%-55%; durable immune responses; mainly grade 1-2 AEs	Antigen presentation → T-cell activation	[136-138]
NCT03175172 (CRS-207 + Pembrolizumab, 2020 Cancers)	Phase II	Small cohort (not disclosed)	Live-attenuated Listeria-based MSLN vaccine + PD-1 inhibitor	No significant clinical activity; trial terminated	MSLN vaccine + PD-1 blockade	ClinicalTrials.gov
NCT00327652/NCT00358945 (CRS-207 + Chemo, 2007-2012)	Phase I/Ib	*n* = 18 + 35	CRS-207 combined with pemetrexed/platinum chemo	DCR: 89%; no OS improvement in later expansion	Vaccine-induced immune priming + chemo	ClinicalTrials.gov
NCT02879669 (ONCOS-102, 2019 J Immunother Cancer)	Phase I	*n* = 12	GM-CSF–expressing oncolytic adenovirus + chemo	ORR: 30%; DCR: 90%; mPFS: 9.8 months; mOS: 25.0 months (*vs.* 13.5 control)	Oncolysis + T-cell infiltration enhancement	[143-145]
NCT01119664 (Ad-IFN, 2014-2018 series)	Phase I	*n* = 21	Intrapleural adenoviral IFN-β gene therapy	DCR: 67%; mOS: 21.5 months; manageable safety	Local IFN signaling → immune activation	[139-142]
NCT01721018 (HSV1716, 2018 Gene Therapy)	Pilot	*n* = 21	Intrapleural oncolytic HSV1716	ORR: 0%; 50% stable disease; immune activation observed	Oncolytic HSV → local immune stimulation	[143,147-149]
NCT03546426 (MESOVAX)/NCT05765084 (Immuno-MESODEC, 2022-2023 conference)	Phase I/II	MESOVAX *n* = 15; Immuno-MESODEC planned *n* = 36	Autologous DC vaccine + Pembrolizumab WT1mRNA-electroporated DC + Atezolizumab + chemo	NCT03546426: ORR: ~56% (BICR); mPFS: 6.7 months; mOS: 20.4 months NCT05765084: Ongoing; immune activation signals reported; survival data pending	DC activation + PD-1 blockade WT1 antigen presentation + PD-L1 inhibition	[99,135] ClinicalTrials.gov

DC: Dendritic cell; ICI: immune checkpoint inhibitor; mOS: median overall survival; AEs: adverse events; MSLN: mesothelin; PD-1: programmed cell death protein 1; DCR: disease control rate; GM-CSF: granulocyte-macrophage colony-stimulating factor; ORR: objective response rate; mPFS: median progression-free survival; IFN-β: interferon beta; HSV1716: Oncolytic Herpes Simplex Virus type 1 strain 1716; BICR: blinded independent central review; PD-L1: programmed death-ligand 1.

Across cellular immunotherapy strategies, mesothelin (MSLN)-directed platforms remain the dominant target. Early-phase CAR-T studies demonstrate modest ORR (12%-26%) with limited durability data, whereas armored or PD-1–resistant constructs (e.g., NAC-T) show substantially higher response rates in small cohorts (ORR 63.6%; mOS 25.6 months), suggesting that overcoming intratumoral immune suppression is critical. TRuC-T therapy demonstrates measurable activity with defined median PFS (5.6 months) and OS (11.2 months), providing the most mature survival data among MSLN-targeted cellular therapies to date.

The most mature evidence comes from MSLN-directed CAR-T cells. The landmark phase I/II trial conducted at Memorial Sloan Kettering Cancer Center (MSKCC) (NCT02414269) evaluated intrapleural infusion of inducible caspase 9 (iCasp9)-mesothelin-targeting CAR with CD28 and CD3ζ signaling domains (M28z) CAR-T cells - incorporating a CD28 co-stimulatory domain and a safety “suicide switch” - followed by systemic pembrolizumab. Among 21 heavily pretreated patients (including 19 with MPM), this regimen produced unprecedented outcomes for relapsed disease, with a median OS of 23.9 months, two complete metabolic responses, and durable CAR-T persistence beyond 100 days in nearly 40% of patients. Correlative studies demonstrated increased TILs and reduced markers of exhaustion following PD-1 blockade, providing mechanistic support for sequential combination ICI therapy and establishing a blueprint for CAR-T–ICI integration in MPM^[109,114,115]^. By supplying large numbers of tumor-specific effector cells directly into the pleural cavity, cellular immunotherapies circumvent the T-cell exclusion and suppressive cellular barriers (Tregs, MDSCs) detailed in Section MECHANISTIC INSIGHTS INTO THERAPEUTIC ACTION, reinforcing the rationale for combining CAR-T cells with checkpoint inhibitors to sustain anti-tumor immunity. Next-generation MSLN-directed platforms aim to improve T-cell persistence, reduce immunogenicity, and overcome the profoundly suppressive pleural TME. The phase I study of human CAR modified T cells in patients with mesothelin expressing cancers (NCT03054298), employing a fully human scFv to minimize anti-murine immune responses, has demonstrated feasible expansion and sustained persistence in early-phase studies, with a favorable safety profile. ATA2271 (NCT04577326) represents a further evolution of this concept: these CAR-T cells incorporate an undetermined signaling backbone and an intrinsic PD-1 dominant-negative receptor to resist exhaustion. Early findings show robust expansion and persistence in low-dose cohorts, although a fatal serious adverse event (SAE) in the high-dose cohort underscores the importance of careful dose optimization in future development^[116-118]^.

Beyond MSLN, several trials have explored alternative antigenic targets relevant to the unique stromal and mesothelial biology of MPM. The FAP-CAR-T trial (NCT01722149) targeted fibroblast activation protein (FAP), a key mediator of the dense fibrotic matrix characteristic of MPM. Despite the small sample size (*n* = 3), the study demonstrated intratumoral CAR-T expansion and survival beyond 18 months in two patients, highlighting the potential of stromal-directed strategies to remodel the TME and reverse immune exclusion. Similarly, podoplanin (PDPN)-CAR-T therapy (NCT03618381), targeting PDP - another clinically relevant mesothelial antigen - has entered phase I evaluation, with ongoing analyses focused on trafficking, biodistribution, and safety^[115,119-122]^.

Complementing traditional CAR-T constructs, the TCR fusion construct T (TRuC-T) cell platform represents an innovative approach that integrates CAR specificity into the native TCR complex. In the phase I trial of gavo-cel (NCT03585764), 39 patients with advanced MPM received TRuC-T cells targeting MSLN, achieving an ORR of 21%, median PFS of 5.6 months, and median OS of 13.7 months. These findings highlight the enhanced signaling potency and physiologic activation afforded by TRuC designs, offering a potentially superior alternative to conventional second-generation CAR-T architectures. To further counteract CAR-T exhaustion within the immunosuppressive MPM microenvironment, MSLN-targeted CAR-T cells armored with an anti-PD-1 nanobody [nanobody-armored CAR-T cells (NAC-T)] were developed. In a first-in-human study involving 11 patients with refractory malignant mesothelioma, intravenous infusion of 5 × 10^6^-20 × 10^6^ cells/kg NAC-T achieved an ORR of 63.6%, DCR of 100%, median PFS of 5.0 months, and median OS of 25.6 months, without dose-limiting toxicity. Single-cell and TCR sequencing demonstrated clonal expansion of tumor-reactive T-cell subsets, underscoring the therapeutic potential of locally enhanced checkpoint blockade within engineered cellular platforms. Beyond adaptive T-cell engineering, nanotechnology-based approaches are emerging to reprogram innate immunity and metabolic vulnerabilities within the MPM microenvironment. Recent evidence indicates that MPM-associated pleural effusions exhibit selenium deficiency and reduced selenoprotein expression [e.g., selenoprotein P (SEPP1)], correlating with impaired natural killer cell (NK-cell) function. To address this redox imbalance, lentinan-functionalized selenium nanoparticles (LET-SeNPs) were developed as immunomodulatory agents capable of restoring bioavailable selenium locally. Mechanistically, LET-SeNPs activate the Thioredoxin reductase 1–phosphorylated signal transducer and activator of transcription 3 (TrxR1–pSTAT3) axis, upregulate natural killer group 2, member D (NKG2D) expression, and enhance NKG2D–NKG2D ligands (NKG2DL)–mediated cytotoxicity. Preclinical spheroid and organoid models demonstrated improved NK-cell infiltration, reduced invasive capacity, and decreased matrix metalloproteinase-2 (MMP-2) expression. Together, these findings suggest that metabolic trace-element reprogramming may synergize with engineered T-cell strategies to reinforce multi-layered immune control in MPM^[123-129]^.

Collectively, the expanding portfolio of adoptive cellular therapies in MPM reflects a multipronged attempt to overcome the immunologic bottlenecks that limit ICI efficacy: supplying effector cells to overcome low T-cell infiltration, engineering exhaustion-resistant phenotypes, targeting both tumor and stromal antigens to dismantle physical and biochemical barriers, and integrating CAR-T with ICIs to sustain antitumor immunity. As translational advances continue to refine antigen selection, delivery strategies, and resistance-proof CAR architectures, cellular immunotherapy is poised to play a central role in next-generation therapeutic combinations designed to surmount the deeply entrenched immune resistance landscape of MPM^[130-132]^.

A fundamental contributor to primary resistance to ICIs in MPM is the failure of adequate antigen presentation, which results in insufficient priming and expansion of tumor-reactive T cells. DC vaccination seeks to directly correct this deficit by *ex vivo* generation of professional antigen-presenting cells. This approach typically involves isolating autologous monocyte-derived DC precursors, maturing them with defined adjuvants, and pulsing them with tumor-associated antigens - either whole tumor lysate or specific antigens such as Wilms tumor 1 (WT1) - before reinfusion. Upon administration, these antigen-loaded DCs traffic to secondary lymphoid organs, where they efficiently prime naïve CD4^+^ and CD8^+^ T cells, generating a broad, polyclonal, and durable antitumor immune response capable of synergizing with ICIs^[133-136]^.

The ongoing phase II/III dendritic cell immunotherapy for mesothelioma (DENIM) trial (NCT03610360) is one of the key ongoing clinical studies evaluating this strategy in MPM. As a randomized, placebo-controlled study testing tumor-lysate–pulsed DC vaccination as maintenance therapy following first-line chemotherapy, DENIM builds on earlier phase I/II signals in which autologous DC vaccination produced durable antigen-specific immunity and median OS reaching up to 27 months in selected cohorts. Parallel early-phase studies now explore rational DC–ICI combinations. The pembrolizumab plus autologous dendritic cell vaccine in patients with PD-L1 negative advanced mesothelioma who have failed prior therapies (MESOVAX) phase I trial (NCT03546426) evaluates autologous DC vaccination administered concurrently with pembrolizumab in previously treated MPM, aiming to enhance T-cell priming while preventing subsequent PD-1–mediated exhaustion. Initial data indicate that the regimen is feasible, immunogenic, and capable of inducing disease stabilization in a subset of patients. In frontline disease, the Immuno-mesothelioma dendritic cell (MESODEC) trial (NCT05765084) is testing a triplet strategy combining WT1-targeted DC vaccination with atezolizumab and standard chemotherapy, leveraging chemotherapy-induced ICD, WT1-specific T-cell activation, and PD-L1 blockade to maximize priming and effector maintenance. Collectively, these trials reflect a mechanistically coherent development path in which DC vaccines augment antigen presentation while ICIs sustain effector functionality within the chronically immunosuppressive MPM microenvironment, supporting their integration into multimodal immunotherapeutic strategies^[26,40,135,137-139]^.

### Oncolytic viruses to convert “cold” to “hot” tumors

A major obstacle to effective immunotherapy in MPM is the predominance of immunologically “cold” tumors, characterized by limited T-cell infiltration and weak antigenicity. Oncolytic viruses offer a compelling strategy to overcome this barrier by acting as *in situ* vaccines: they selectively infect tumor cells, induce ICD, and release a diverse array of neoantigens, DAMPs, and viral PAMPs that ignite innate and adaptive immune responses. The resulting interferon beta (IFN-β) enhances dendritic-cell activation, promotes chemokine-driven T-cell recruitment, and primes de novo antitumor immunity - thereby establishing a more permissive microenvironment for subsequent ICI therapy^[133,140-143]^.

The GM-CSF–expressing adenovirus a randomised open-label phase I/II study adding ONCOS-102 to pemetrexed/cisplatin in patients with unresectable malignant pleural mesothelioma (ONCOS-102) (NCT02879669) has demonstrated these immunostimulatory properties in early-phase clinical studies. In combination with chemotherapy, ONCOS-102 markedly increased intratumoral CD8^+^ T-cell infiltration and upregulated immune-activation gene signatures, with the most pronounced immunologic changes correlating with clinical benefit. Notably, exploratory follow-up analyses revealed upregulation of PD-L1 after ONCOS-102, providing a mechanistic rationale for combining or sequencing with PD-1 inhibitors. Early clinical experiences using ONCOS-102 followed by pembrolizumab reported sustained T-cell expansion and durable tumor control, supporting a synergistic “prime-and-boost” paradigm wherein oncolytic viruses initiate immune activation and ICIs maintain effector function^[144-146]^.

Beyond adenoviral platforms, the oncolytic herpesvirus HSV1716 (Oncolytic Herpes Simplex Virus type 1 strain 1716; NCT01721018) has shown favorable safety and an ability to stimulate localized immune activation in MPM. This lays the groundwork for future strategies combining herpes simplex virus (HSV)-based therapy with PD-1/PD-L1 blockade. Preclinical studies further reinforce these concepts: the cytokine-armed adenovirus Ad5/3-E2F-D24-hTNFα-IRES-hIL-2 (TILT-123), encoding TNF-α and IL-2, synergized with anti–PD-1 therapy in mouse models, inducing systemic “abscopal-like” tumor regression and durable immunologic memory. These findings highlight the broader potential of oncolytic immunotherapy to reprogram the mesothelioma microenvironment, thereby converting inherently cold tumors into ICI-responsive lesions^[147-151]^.

### Emerging biological pathways and targetable regulators of resistance

Beyond the more established mechanisms discussed above, several foundational biological pathways are increasingly recognized as critical contributors to ICI resistance in MPM, presenting a rich landscape for novel therapeutic intervention^[27]^.

Genomic Determinants: Specific somatic alterations shape the immune contexture: CDKN2A deletion, one of the most common genomic events in MPM, has been associated with intrinsic resistance to PD-1 blockade, potentially through the suppression of interferon signaling pathways that are crucial for antigen presentation. In contrast, BAP1 loss, another frequent occurrence, is paradoxically linked to a more “inflamed” TME characterized by increased immune infiltration and may predict enhanced ICI sensitivity in certain contexts, highlighting the complex interplay between driver mutations and anti-tumor immunity^[152]^.

Metabolic Immunosuppression: Multiple metabolic pathways beyond arginine contribute to the immunosuppressive TME. These include lactate accumulation from aerobic glycolysis, which acidifies the TME and directly impairs T-cell effector function and cytokine production; indoleamine 2,3-dioxygenase 1 (IDO1)-mediated tryptophan depletion, which activates immunosuppressive pathways in T cells; and the activation of the potent adenosine pathway, where the ectoenzymes CD39 and CD73 generate extracellular adenosine that suppresses T cells and NK cells via the Adenosine A2A receptor^[27,153-155]^.

Wingless-related integration site (Wnt)/β-catenin Signaling: The aberrant activation of this developmental pathway is a recognized non-mutational driver of T-cell exclusion in many cancers, including a subset of MPM. It prevents the recruitment of CD103^+^ DCs and effector T cells into the tumor, creating an “immune-desert” phenotype^[156-159]^.

TGF-β Signaling: TGF-β is a master regulator of the fibrotic, immune-suppressed TME in MPM. It is a potent inducer of Treg differentiation and a key activator of CAFs, which drive the deposition of a collagen-rich extracellular matrix that physically excludes T cells. This pathway is now being actively targeted in the clinic, for example, with the TGF-β inhibitor OT-101 in combination with pembrolizumab (NCT05425576)^[160-163]^. Inhibition of TGF-β signaling reverses the fibrotic stromal barrier and Treg accumulation highlighted in Section MECHANISTIC INSIGHTS INTO THERAPEUTIC ACTION, representing a rational strategy to remodel the TME and enhance ICI efficacy.

Multiple immunotherapeutic platforms are currently being explored in MPM. To date, dual immune checkpoint blockade has demonstrated a statistically significant OS improvement in a phase III setting. This level of evidence offers a comparative context for interpreting the clinical development of other investigational approaches. Metabolic interventions, such as arginine depletion in ASS1-deficient tumors, illustrate the promise of biomarker-driven stratification but have yet to produce consistent survival gains at the population level. Their efficacy appears context-dependent and vulnerable to adaptive metabolic rewiring. Similarly, MSLN-targeted armored CAR-T constructs generate striking early response rates and encouraging survival signals; however, these findings derive from small, non-randomized cohorts and require validation in adequately powered trials.

Regional approaches, including tumor treating fields (TTFields), produce survival outcomes comparable to historical chemotherapy standards but lack direct comparison with immune checkpoint blockade. At the preclinical level, immunometabolic reprogramming strategies, including nanoparticle-mediated restoration of natural killer cell cytotoxicity, underscore the mechanistic sophistication of the field, yet remain translationally immature.

Nevertheless, several significant limitations must be taken into account. The majority of combination strategies for MPM are still in early-phase clinical development, often relying on small, non-randomized cohorts without biomarker-driven patient stratification. Consequently, the reported response signals may be more reflective of selection bias rather than genuine mechanistic synergy. In addition, overlapping toxicities, high costs, and logistical complexities - especially for cellular and regionally delivered therapies - present formidable translational hurdles. Currently, many combination approaches are empirically constructed based on biological plausibility rather than prospectively validated resistance mechanisms. Future progress will hinge on rigorous randomized validation, adaptive trial designs, and robust biomarker integration to differentiate rational synergy from mere additive experimentation. Moving forward, real progress will hinge not just on developing new biological concepts, but on integrating robust biomarkers, designing smarter combinations, and confirming durable survival benefits through rigorous randomized trials - rather than relying on early-phase signals^[129,164,165]^.

## BIOMARKERS AND PRECISION IMMUNO-ONCOLOGY IN MPM

### From empiricism to precision

MPM has historically been managed with empiric therapeutic strategies, resulting in consistently poor outcomes across its diverse histologic and molecular subtypes. The introduction of ICIs, specifically anti–PD-1 and anti–CTLA-4 antibodies, has marked the beginning of a new therapeutic epoch. Nonetheless, despite transformative benefits in certain patient subgroups, overall response rates remain modest - approximately 23%-25% for monotherapy and 35%-40% for dual immunotherapy combinations, as demonstrated in the CheckMate 743 and MAPS2 trials^[166]^.

**Figure 4:**
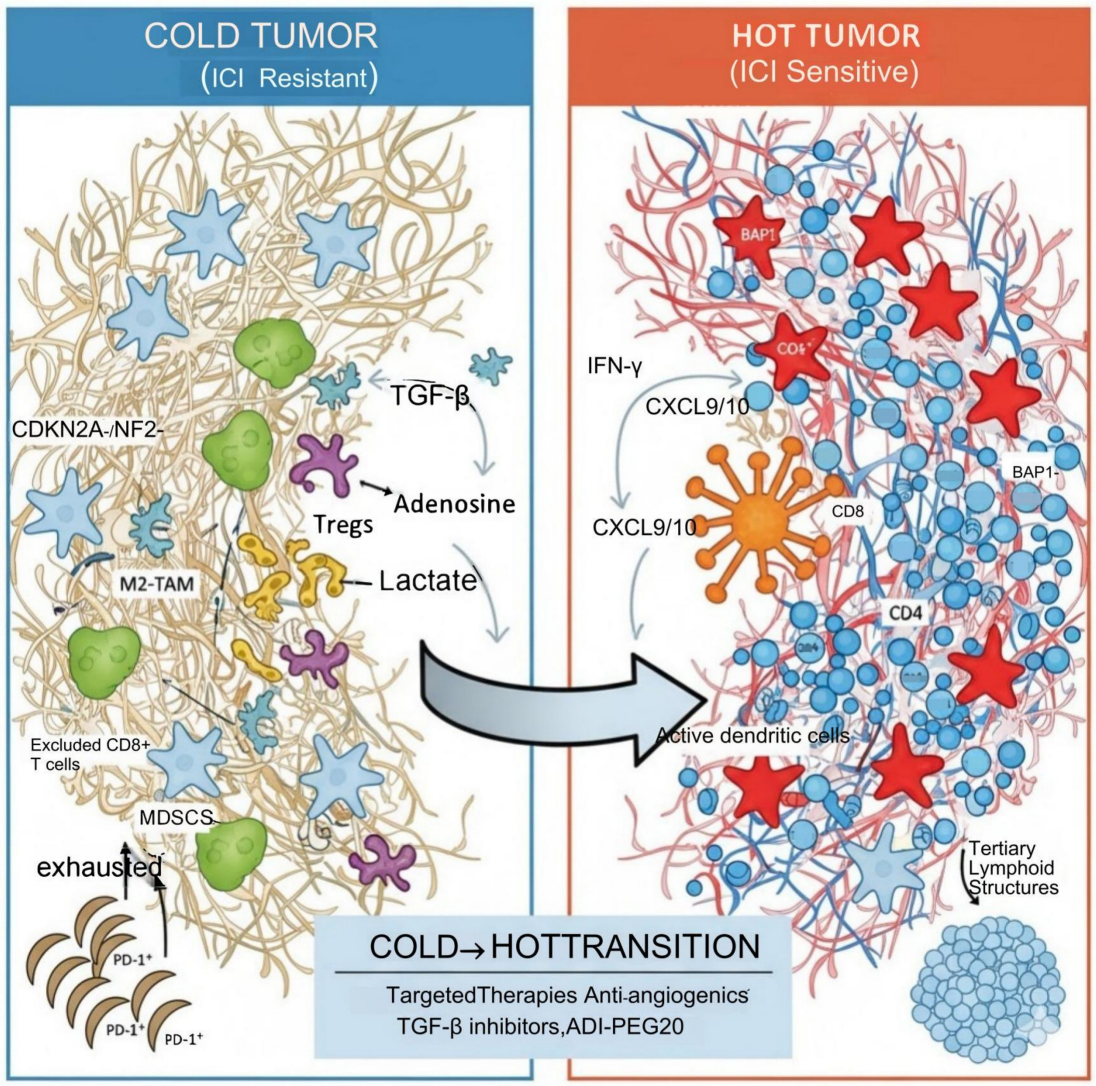
MPM TME: from “cold” to “hot”. MPM: Malignant pleural mesothelioma; TME: tumor microenvironment; ICI: immune checkpoint inhibitor; CDKN2A: cyclin-dependent kinase inhibitor 2A; NF2: neurofibromin 2; TGF-β: transforming growth factor beta; Tregs: regulatory T cells; M2-TAM: M2-type tumor-associated macrophage; CD8^+^ T cells: cytotoxic T lymphocytes; MDSCs: myeloid-derived suppressor cells; PD-1: programmed cell death protein 1; IFN-γ: interferon γ; CXCL9/10: chemokine (C-X-C motif) ligand 9/10; BAP1: BRCA1-associated protein 1; ADI-PEG20: PEGylated arginine deiminase.

This limited and heterogeneous clinical efficacy underscores the pressing need for predictive and prognostic biomarkers to optimize patient selection, treatment sequencing, and rational combination therapy design, as summarized in the integrative framework in Figure 4. Unlike melanoma or non-small cell lung cancer (NSCLC), MPM is characterized by a low TMB, a restricted neoantigen landscape, and a highly complex TME dominated by immune exclusion and stromal components - all of which pose significant challenges to biomarker development^[167-169]^.

### Established biomarkers: PD-L1, TMB, and histologic context

PD-L1 expression, although widely assessed, demonstrates inconsistent predictive utility in MPM. In the CheckMate 743 trial, PD-L1 expression ≥ 1% was correlated with improved OS in the nivolumab–ipilimumab group (median OS 18.0 *vs.* 13.3 months; HR = 0.69); however, clinical benefit was also observed in PD-L1–negative patients, indicating its limited discriminatory power. Furthermore, inter-assay variability (e.g., between the PD-L1 IHC 22C3 and PD-L1 IHC SP263 antibody clones) and significant intratumoral heterogeneity further compromise the reliability of PD-L1 as a standalone biomarker^[170,171]^.

Similarly, TMB in MPM is generally low, typically below 2 mutations/Mb, which aligns with its non-mutagenic, asbestos-driven etiology. As a result, TMB alone is inadequate for predicting ICI responsiveness. However, specific mutational signatures - such as those associated with defective DNA damage repair (DDR) and alterations in chromatin-remodeling genes [e.g., *BAP1*, *NF2*, SET domain containing 2 (*SETD2*)] - may hold greater immunologic relevance^[172-174]^.

Histologic subtype remains the most clinically actionable biomarker to date. Non-epithelioid MPM, despite its historically unfavorable prognosis, derives disproportionate benefit from dual ICI regimens (e.g., in CheckMate 743: OS 18.1 *vs.* 8.8 months; HR = 0.46), likely attributable to higher baseline immune infiltration and inflamed transcriptional profiles^[175,176]^.

### Genomic and transcriptomic biomarkers

Beyond conventional markers, comprehensive genomic profiling has uncovered a spectrum of mutations and transcriptional phenotypes that critically influence immune responsiveness.

BAP1 loss, occurring in 50%-60% of MPM cases, promotes an “inflamed” tumor phenotype, characterized by enhanced interferon γ (IFN-γ) signaling, elevated chemokine (C-X-C motif) ligand 9/10 (CXCL9/CXCL10) expression, and increased infiltration of cytotoxic lymphocytes. Retrospective analyses indicate improved response rates to ICIs in BAP1-deficient tumors (e.g., as seen in NCT01773655 and correlative studies from CheckMate 743)^[177]^.

Conversely, CDKN2A deletion - found in approximately 70% of cases - is associated with resistance to PD-1 blockade, likely mediated through impaired interferon signaling and downregulation of MHC-I expression. Similarly, dysregulation of the NF2/Hippo pathway contributes to immune evasion via YAP-mediated induction of PD-L1 expression and suppression of pro-inflammatory cytokines^[178-181]^.

Transcriptomic classifications have further refined the distinction between “hot” and “cold” immune phenotypes. Tumors exhibiting enriched immune gene signatures - including elevated expression of chemokine (C-X-C motif) ligand 13 (CXCL13), granzyme (*GZMB*), and human leukocyte antigen (*HLA*) genes - correlate with improved OS and greater ICI responsiveness^[152,182]^.

### Epigenetic and metabolic biomarkers

Emerging evidence underscores the role of epigenetic dysregulation as both an oncogenic driver and a key determinant of immunogenicity. BAP1 loss, SETD2 mutations, and enhancer of zeste homolog 2 (EZH2) overexpression collectively establish a repressive chromatin state that suppresses antigen presentation. Pharmacologic intervention with HDAC or EZH2 inhibitors (e.g., tazemetostat, NCT02860286) can restore MHC-I expression and promote immune infiltration, highlighting epigenetic modulation as a promising biomarker-guided therapeutic avenue^[183-186]^.

On the metabolic front, arginine metabolism has arisen as a critical regulator of immunotherapy response. ASS1-deficient tumors display arginine auxotrophy, initiating a metabolic competition with infiltrating T cells for essential nutrients. Clinical trials such as ATOMIC-Meso (NCT02709512) have demonstrated that systemic arginine depletion via ADI-PEG20 not only targets tumor metabolism but also reprograms the TME to favor anti-tumor immunity. Other metabolic pathways - including lactate accumulation, IDO activation, and adenosine signaling - have also been implicated in immune suppression and may serve as composite metabolic biomarkers^[187-191]^.

### Circulating biomarkers and liquid biopsy approaches

Liquid biopsy technologies enable noninvasive, longitudinal biomarker assessment through multiple complementary approaches. Soluble PD-L1 levels in plasma correlate with tumor PD-L1 expression and poorer prognosis in retrospective analyses. Meanwhile, circulating tumor DNA and exosomal RNA facilitate dynamic monitoring of mutational burden and immune-related gene expression patterns, including interferon response signatures. Additionally, peripheral immune profiling - particularly an elevated baseline CD8^+^/Treg ratio and expanded TCR clonality - shows association with improved survival in patients undergoing ICI therapy, as demonstrated in the NCT04577326 translational substudy. These integrated liquid biopsy modalities are currently undergoing prospective validation in ongoing trials such as MESOTHRIVE (NCT04990777) and IMMUNO-MESO (NCT05765084)^[192-195]^.

### Integrative and artificial intelligence-driven biomarker discovery

Artificial intelligence (AI)–driven digital pathology is emerging as a critical enabler of precision stratification. Deep learning models applied to whole-slide images can achieve expert-level tumor detection and grading while inferring molecular features such as microsatellite instability (MSI) status, PD-L1 expression, and oncogenic mutations directly from routine histology. Integrative AI frameworks combining histomorphology, multi-omics, radiomics, and clinical data may facilitate dynamic risk prediction and personalized treatment allocation.

However, translation into routine practice will require prospective validation, standardized data harmonization, interpretability improvements, and regulatory alignment. Ultimately, the convergence of engineered immunotherapies, nanomedicine-based microenvironment modulation, and AI-guided patient stratification may redefine the therapeutic landscape of MPM, shifting from empiric management toward adaptive, biology-driven precision oncology^[196-199]^.

### Clinical translation and future directions

Despite notable advances, the translation of biomarkers into routine clinical practice confronts several obstacles: inter-assay variability, limited tissue availability, and small, histologically heterogeneous trial cohorts. Looking ahead, adaptive biomarker-guided trials - exemplified by the platform design of PRISM-Meso (NCT05022634) - aim to stratify patients in real time based on immune-genomic characteristics. Moreover, AI-driven predictive models that integrate radiomic features with molecular signatures (i.e., radiogenomics) offer promising avenues for noninvasive biomarker prediction.

Ultimately, the convergence of molecular precision, immunologic insight, and computational analytics is poised to redefine MPM management, steering the field away from empirical immunotherapy toward a future of truly personalized immuno-oncology^[200,201]^.

## CONCLUSION

MPM remains a biologically aggressive and therapeutically refractory malignancy. Although immune checkpoint blockade - most notably nivolumab plus ipilimumab, as established in CheckMate 743 (median OS 18.1 months) - has reshaped first-line management, durable clinical benefit remains limited to a subset of patients, reflecting the layered, adaptive, and spatially organized mechanisms of immune resistance within the pleural TME.

A mechanism-guided therapeutic framework is therefore critical. T-cell exhaustion, NK-cell dysfunction, metabolic liabilities such as arginine auxotrophy, stromal fibrosis, aberrant angiogenesis, and dysregulated pathways including TGF-β and Hippo signaling collectively generate an immunosuppressive niche that constrains therapeutic efficacy. These vulnerabilities have catalyzed the development of multidimensional strategies, including exhaustion-resistant and armored CAR-T/TRuC-T platforms, stromal- and antigen-targeted cellular therapies, metabolic deprivation approaches (e.g., ADI-PEG20 in ASS1-deficient tumors), epigenetic modulation to restore antigen presentation, vascular normalization strategies, and biomaterial-enabled locoregional delivery systems. Emerging nanotechnology-based interventions - such as selenium nanoparticle–mediated redox reprogramming to enhance NK-cell cytotoxicity, recently investigated in Chinese translational studies - further exemplify how metabolic and innate immune modulation may synergize with engineered cellular platforms. Regional modalities including TTFields have also demonstrated encouraging survival benefits without added systemic toxicity in phase II evaluation, suggesting that mitotic stress–induced tumor immunogenicity may complement immunotherapeutic strategies.

Concurrently, advances in AI–assisted histopathology, spatial transcriptomics, multi-omics integration, and liquid biopsy technologies are refining biomarker discovery and patient stratification. The convergence of spatial biology and computational modeling holds promise for aligning specific resistance mechanisms with rationally selected combinatorial regimens in a precision oncology framework.

Looking forward, sustained progress will depend on biomarker-enriched and adaptively designed clinical trials, deeper integration of immunology, bioengineering, and computational sciences, and broader geographic representation of enrolled populations. While pivotal trials have been conducted predominantly in Western cohorts, expanding high-quality clinical and translational studies in Asian populations - including cellular and nanotechnology-based immunotherapies - will be essential to validate biomarker-driven strategies across diverse genetic and environmental backgrounds. Linking mechanism to target and to therapeutic strategy provides a coherent translational roadmap and offers the most credible path toward achieving durable immune control in this surface-spreading malignancy^[86,202-206]^.

## DECLARATIONS

### Copyright

© The Author(s) 2026.
